# Repair of a Large Congenital Peritoneopericardial Diaphragmatic Hernia with Canine Small Intestinal Submucosa Patch Reinforcement in a Dog: A Case Report

**DOI:** 10.3390/ani16193048

**Published:** 2026-09-28

**Authors:** Tae-Seong Moon, Sung-Yeon Moon, Su-Han Woo, Eun-Ji Jeong, Chang-Min Park, A-Jin Lee, Hwi-Yool Kim, Jung-Moon Kim

**Affiliations:** Department of Veterinary Medicine, College of Veterinary Medicine, Konkuk University, Seoul 05029, Republic of Korea; xotjd6990@naver.com (T.-S.M.); hykim@konkuk.ac.kr (H.-Y.K.)

**Keywords:** canine, peritoneopericardial diaphragmatic hernia, PPDH, small intestinal submucosa, SIS, diaphragmatic defect, biomaterial, surgical repair

## Abstract

Peritoneopericardial diaphragmatic hernia (PPDH) is a congenital anomaly allowing communication between the peritoneal and pericardial cavities. Surgical repair of large diaphragmatic defects can be challenging due to insufficient tissue for primary closure. A 2-year-old intact female White Miniature Schnauzer weighing 6.23 kg presented with a 1-month history of panting and exercise intolerance. Thoracic radiography, ultrasonography, echocardiography, and CT confirmed PPDH with herniation of the left medial and quadrate liver lobes into the pericardial sac. Surgical repair via midline celiotomy revealed a large defect (4.5 × 5 cm) involving approximately 70% of the central tendon. Primary closure was incomplete because only two simple interrupted sutures could be placed at the dorsal margin due to excessive tension, leaving a triangular residual defect larger than 3 × 3 cm at the ventral aspect, which was closed with a two-layer canine small intestinal submucosa (cSIS) patch applied over the residual defect. Reherniation occurred 42 days postoperatively at the margin between the diaphragm and the cSIS patch. At the second surgery, grossly observed tissue presumed to be newly formed self-tissue at the cSIS site enabled direct suture repair. No recurrence was observed during a 5-month follow-up, and a telephone interview with the owner 3 years after the second surgery confirmed that the dog remained free of clinical signs. This case suggests that cSIS may serve as a feasible reinforcement biomaterial for large diaphragmatic defects where primary closure alone is insufficient, although conclusions drawn from a single case remain preliminary, and surveillance for marginal reherniation is warranted.

## Simple Summary

A dog had a birth defect called a peritoneopericardial diaphragmatic hernia (PPDH), where a hole in the diaphragm allowed part of the liver to move into the sac surrounding the heart. This caused breathing difficulty and reduced exercise tolerance. During surgery, the hole was too large to close with stitches alone, so a patch made from the intestinal lining of another dog (canine small intestinal submucosa, or cSIS) was used to reinforce the repair. Six weeks later, the hernia recurred at the edge where the patch met the dog’s own tissue, but the patch itself had not broken down. At the second surgery, tissue that appeared to be the dog’s own new tissue had formed at the patch site, allowing a simpler repair; however, this was not confirmed with a tissue biopsy. Five months later, the dog remained healthy with no recurrence. Three years after the surgery, a phone call to the owner confirmed that the dog was still doing well and had no signs of illness. Because this report describes only one dog, these findings are preliminary and cannot be generalized; still, this experience suggests that cSIS may be a helpful option for reinforcing large diaphragmatic holes, and owners and veterinarians should monitor for reherniation at the patch edges during the healing period.

## 1. Introduction

Peritoneopericardial diaphragmatic hernia (PPDH) is a congenital malformation characterized by persistent communication between the peritoneal cavity and the pericardial sac, resulting from failure of the septum transversum to fuse properly with the pleuroperitoneal membranes during embryonic development [1]. PPDH is the most common congenital pericardial anomaly in dogs and cats [2,3]. While the true prevalence is low—estimated at 0.015% of hospitalized dogs—the condition may be underdiagnosed because some affected animals remain asymptomatic throughout their lives [4,5].

Clinical signs of PPDH are variable and depend on the type and amount of herniated abdominal organs, the degree of cardiopulmonary compression, and the presence of incarceration. The most common presenting signs include respiratory signs (dyspnea, tachypnea, panting) and gastrointestinal signs (vomiting, anorexia, diarrhea), with respiratory signs being more prevalent in cats and gastrointestinal signs in dogs [2,3]. Attenuation or muffling of heart sounds is a characteristic physical examination finding resulting from displacement of the heart by herniated abdominal organs [6,7].

Diagnosis of PPDH is primarily achieved through diagnostic imaging. Thoracic radiography typically reveals a markedly enlarged cardiac silhouette, dorsal displacement of the trachea, and obscuration of the diaphragmatic border [1,2]. Abdominal ultrasonography and echocardiography allow direct visualization of herniated organs and assessment of cardiac structure and function [7,8]. Computed tomography (CT) provides detailed characterization of the diaphragmatic defect and aids in surgical planning [9,10].

Surgical repair is the treatment of choice for animals with clinical signs attributable to PPDH [2,4]. A multi-institutional study of 128 dogs reported that 97% of surgically treated dogs were discharged from the hospital; long-term follow-up was available for 87 dogs at a median of 1062 days, with no hernia recurrence reported in any surgically treated dog [5]. Primary closure by direct apposition of the diaphragmatic margins is the preferred technique; however, large defects that constitute a substantial portion of the diaphragm may preclude tension-free closure. In such cases, alternative techniques including pericardial flap reconstruction, ventral diaphragmatic advancement, muscle flap transposition, or biomaterial patch implantation may be required [11,12].

Small intestinal submucosa (SIS) is an acellular extracellular matrix biomaterial that has been reported to promote site-specific host tissue remodeling and neovascularization while providing temporary mechanical support during tissue regeneration. Both porcine SIS (pSIS) and canine SIS (cSIS) have been used for hernia repair in dogs; however, pSIS has been more extensively studied and is commercially available, whereas cSIS remains non-commercialized [13,14]. An important theoretical immunological distinction exists between these graft types: pSIS contains the Galα(1,3)Gal (α-Gal) epitope distributed transmurally throughout the scaffold, which can potentially elicit a xenogeneic immune response in non-porcine recipients [15], whereas cSIS—as an allogeneic graft in canine patients—is inherently devoid of this antigenic burden [16]. Despite this theoretical consideration, clinical evidence for cSIS remains limited to a single report of perineal herniorrhaphy in two dogs without signs of rejection or recurrence over 12 months [14]. To the authors’ knowledge, the use of cSIS for reinforcement of large PPDH defects in dogs has not been previously reported. This case report describes the surgical management of a large congenital PPDH using primary repair combined with cSIS patch reinforcement, the occurrence of marginal reherniation, and subsequent successful re-repair utilizing presumed self-tissue formed at the cSIS application site.

## 2. Case Presentation

A 2-year-old intact female White Miniature Schnauzer weighing 6.23 kg was referred to the Konkuk University Veterinary Medical Teaching Hospital for evaluation of a 1-month history of panting, excitement, and exercise intolerance during walks. The owner reported that the clinical signs had been progressively worsening and were triggered or exacerbated by physical activity. There was no history of trauma, and the dog had been otherwise healthy with no previous surgical or medical conditions. The dog was not receiving any medications at the time of presentation.

On physical examination, the dog’s body temperature was 39.1 °C, heart rate was 132 bpm, and respiratory rate was 48 breaths/min. The most notable finding on auscultation was muffled heart sounds, particularly on the right hemithorax. No other abnormalities were detected on physical examination.

Hematological and serum biochemical analyses were within reference ranges (Table 1). No abnormalities suggestive of hepatic dysfunction, metabolic disease, or other systemic illness were identified.

Thoracic radiography (right lateral and ventrodorsal views) revealed a large, globoid cardiac silhouette with rightward displacement of the cardiac apex (Figure 1). The diaphragmatic border was obscured. These findings were consistent with cardiomegaly with a morphology suspicious for PPDH [1,3].

Abdominal ultrasonography, performed by a single examiner with the dog positioned in dorsal recumbency using a 7.5 MHz transducer, confirmed herniation of a liver lobe into the pericardial sac (Figure 2A). Color Doppler evaluation confirmed the presence of blood flow within the herniated liver lobe (Figure 2B), ruling out vascular compromise or incarceration at the qualitative level; contrast-enhanced ultrasonography, and quantitative Doppler parameters (such as peak systolic velocity and resistive index) were not used or recorded.

Echocardiography revealed a structure with echogenicity consistent with hepatic tissue adjacent to the right heart within the pericardial sac. No evidence of incarceration or adhesion of the herniated liver to the pericardium or cardiac surface was identified. Importantly, the cardiac structure and function were normal, with no congenital cardiac anomalies or functional deficits detected.

To further characterize the extent of the diaphragmatic defect and assess for additional congenital abnormalities, contrast-enhanced thoracic and abdominal CT was performed under general anesthesia. Multiplanar reconstruction (MPR) images in transverse, sagittal, and dorsal planes were generated for surgical planning. CT confirmed the presence of a liver lobe within the pericardial sac, with leftward displacement of the heart by the mass effect of the herniated organs (Figure 3). The craniocaudal extent of the hernia and the diaphragmatic defect location were assessed on sagittal and dorsal MPR images. No other congenital abnormalities, including sternal anomalies or additional abdominal wall defects, were identified.

The dog was premedicated with buprenorphine (10 μg/kg, IM; Renolphan, Hanlim Pharm, Yongin city, Republic of Korea) and midazolam (0.3 mg/kg, IV; Vascam, HaNa Pharm, Seoul city, Republic of Korea). General anesthesia was induced with propofol (6 mg/kg, IV; Anepol, HaNa Pharm, Seoul city, Republic of Korea) and maintained with 2% isoflurane (Forane, ChoongWae Pharma, Gwacheon city, Republic of Korea) in 100% oxygen. Epidural anesthesia was administered using a mixture of bupivacaine (0.5 mg/kg; Bupivacaine 0.5%, HaNa Pharm, Seoul city, Republic of Korea) and buprenorphine (10 μg/kg; Renolphan, Hanlim Pharm, Yongin city, Republic of Korea). Surgical repair was performed via a midline celiotomy extending from the xiphoid to the pubis. Upon abdominal exploration, the left medial liver lobe and the quadrate lobe were confirmed to be herniated through the diaphragmatic defect into the pericardial sac (Figure 4). The herniated liver lobes were carefully reduced back into the abdominal cavity. No adhesions between the herniated liver and the pericardium or cardiac structures were present.

Following reduction of the herniated liver lobes, the diaphragmatic defect was identified at the central tendon of the diaphragm. On CT, the overall dimensions of the diaphragm were 11 × 11 cm on the thoracic surface. The defect measured approximately 4.5 × 5 cm, accounting for approximately 21% of the total diaphragmatic area, and constituting approximately 70% of the central tendon area (Figure 4).

Primary repair of the defect was first attempted by suturing from the dorsal to the ventral margin of the defect. Only two simple interrupted sutures could be placed at the dorsal margin of the defect; further apposition of the ventral aspect was not possible because the large size of the defect and the limited amount of residual central tendon tissue placed excessive tension on the sutures, and the defect therefore could not be completely closed with primary repair alone. Consequently, a residual defect remained at the ventral aspect of the original defect; it was triangular in shape and measured more than 3 × 3 cm.

Therefore, to reinforce the incompletely closed defect, a 5 × 5 cm canine small intestinal submucosa (cSIS) patch was folded in half to create a double-layer (2-ply) graft and sutured over the residual defect (Figure 4). The 2-ply configuration was selected based on the surgeon’s intraoperative judgment that two layers would provide sufficient thickness and strength both to span the residual defect and to reinforce the partially completed primary repair. The cSIS was prepared according to the method validated by Lee et al. [17]. Briefly, jejunal segments were retrieved through the institutional canine tissue donation program. Tissues were harvested, with written informed owner consent, from client-owned adult dogs that were free of systemic disease other than underlying neurological deficits and euthanized for medical reasons unrelated to this study. All specimens were collected within 2 h post-euthanasia and thoroughly rinsed with sterile normal saline.

The mucosa, muscularis, and serosa were removed by mechanical abrasion, and the remaining submucosa was thoroughly rinsed in sterile normal saline and 80% ethanol to remove cell degradation products. The cSIS was stored at 4 °C in sterile normal saline containing antibiotics (lincomycin HCl, vancomycin HCl, and cefotaxime) for 7 days prior to use [17]. The cSIS patch was secured to the surrounding diaphragmatic margins with 4-0 monofilament polyglyconate sutures (Maxon; Covidien, Fridley, MN, USA) in a simple interrupted pattern at 2–3 mm intervals, with each suture bite incorporating the full thickness of the diaphragm; the graft was joined directly to the edge of the residual defect, without overlapping the surrounding native diaphragm, and no additional reinforcing (buttress) sutures were placed. The subcutaneous tissue was closed with 4-0 Maxon sutures (Covidien, USA), and the skin was closed with 3-0 monofilament polyamide sutures (Dafilon; B. Braun, Melsungen, Germany). The surgery lasted 83 min, with a total anesthesia time of 95 min; intraoperative blood loss was estimated at less than 10 mL, and neither thoracic nor abdominal drainage was placed.

The dog recovered from anesthesia uneventfully. Buprenorphine (10 µg/kg, IM) was administered as an analgesic every 8 h for 2 days, and treatment with cephalexin (22 mg/kg) was continued twice per day for 14 days. Postoperative pain was monitored during hospitalization using the short form of the Glasgow composite measure pain scale (GCMPS-SF); pain scores remained low and consistently below the recommended analgesic intervention threshold (6/24) at all assessments, and no rescue analgesia was required. Postoperative hospitalization lasted 3 days, during which the dog was monitored for respiratory compromise, pneumothorax, and other postoperative complications. No significant postoperative complications were observed. Skin sutures were removed 11 days after surgery.

Follow-up thoracic radiography at the time of suture removal demonstrated a normal cardiac silhouette size, with resolution of the previously observed globoid cardiomegaly (Figure 5A). Abdominal ultrasonography confirmed that the cSIS patch was well maintained at the defect site, with no evidence of reherniation (Figure 5B).

At 42 days postoperatively, physical examination was unremarkable; however, thoracic radiography revealed an increase in cardiac silhouette size to a long axis measurement similar to the preoperative value (90.8 mm) (Figure 6A). Abdominal ultrasonography demonstrated herniation of a structure consistent with a liver lobe into the pericardial sac (Figure 6B), with no evidence of adhesion between the herniated tissue and the heart on ultrasonographic evaluation. Based on these imaging findings, reherniation was diagnosed. Because the diagnosis had already been established by radiography and ultrasonography, and because repeating CT would have required a separate anesthetic event in a patient considered to be at relatively high anesthetic risk, CT was not repeated; instead, the anatomy of the recurrence was assessed by direct visualization during the single anesthetic episode required for surgical repair. The dog subsequently underwent a second surgery via midline celiotomy. The anesthesia protocol was identical to that of the first surgery; the second surgery lasted 49 min, with a total anesthesia time of 58 min, intraoperative blood loss was estimated at less than 5 mL, and no drainage device was placed. Upon exploration, a defect was identified at the margin between the native diaphragm and the cSIS application site; the cSIS patch itself was intact and appeared to have been incorporated into the surrounding diaphragmatic tissue, indicating that the reherniation occurred at the patch–host tissue interface rather than through patch breakdown (Figure 6C). Through this marginal defect, a portion of the cranial margin of a liver lobe and omentum had herniated. On gross inspection, tissue presumed to be newly formed self-tissue was observed at the site where the cSIS patch had been previously applied. This tissue was thin but retained sufficient suture-holding integrity; no active vascularization was grossly identified on its surface; and it appeared continuous with the surrounding native diaphragm to the extent that no discernible boundary separated the two. Its cellular composition was not verified histologically. The herniated liver lobe and omentum were reduced, and the marginal defect was repaired by direct suture closure utilizing this presumed self-tissue; unlike at the initial surgery, closure of the marginal defect was achieved without significant tension and without the use of additional graft material. The abdominal wall was closed routinely without complications. Postoperative pain was assessed using the same pain scale; scores again remained below the intervention threshold, and no rescue analgesia was required.

Five months after the second surgery, thoracic radiography revealed a normal cardiac silhouette with no evidence of cardiomegaly (Figure 7A). Abdominal ultrasonography confirmed an intact diaphragm with no evidence of reherniation (Figure 7B). On physical examination, the dog was clinically normal with no recurrence of the previous clinical signs (panting, excitement, or exercise intolerance). No further episodes of reherniation or clinical deterioration were reported during the 5-month follow-up period. In addition, a telephone follow-up conducted 3 years after the second surgery confirmed that the dog was doing well without any clinical signs.

## 3. Discussion

This case report describes the surgical management of a large congenital PPDH in a 2-year-old Miniature Schnauzer using primary repair combined with cSIS patch reinforcement, subsequent marginal reherniation, and successful re-repair using tissue presumed to be self-tissue formed at the cSIS application site. While the findings of a single case cannot be generalized, they illustrate both the potential utility and the limitations of cSIS as a reinforcement biomaterial for large diaphragmatic defects in dogs and may inform future applications.

The diagnostic approach in this case followed a systematic algorithm. Thoracic radiography provided the initial suspicion of PPDH based on the characteristic finding of a large globoid cardiac silhouette with obscured diaphragmatic border [1,2]. Ultrasonography allowed non-invasive confirmation of liver herniation, and color Doppler evaluation provided qualitative confirmation of blood flow within the herniated liver, which was crucial for ruling out hepatic incarceration—a potentially life-threatening complication of PPDH [7]; however, as quantitative Doppler parameters were not recorded in this case, this assessment remained qualitative. Echocardiography was essential for evaluating cardiac structure and function, as PPDH can be associated with concurrent congenital abnormalities in up to 57.1% of affected dogs, most commonly umbilical hernias, abdominal wall hernias, and sternal anomalies [3,4]. The absence of cardiac abnormalities in this case supported the feasibility of surgical repair. CT provided the most comprehensive assessment of the diaphragmatic defect, confirming the herniated organs and ruling out additional congenital abnormalities, and was invaluable for surgical planning [9,10]. At the time of reherniation, however, CT was not repeated before the second surgery: recurrent hepatic herniation had already been confirmed by radiography and ultrasonography, and a separate anesthetic event solely for diagnostic CT was judged to add unnecessary anesthetic risk in a patient considered to be at relatively high anesthetic risk. Consequently, definitive anatomical characterization of the recurrence—including the marginal location of the defect—was obtained by direct intraoperative visualization (Figure 6C) under the single anesthetic episode required for the repair, which arguably constitutes more direct anatomical evidence than preoperative cross-sectional imaging could have provided, although the absence of repeat CT precluded objective preoperative comparison of the defect anatomy between the first and second surgeries.

Primary closure is the standard technique for PPDH repair when the diaphragmatic defect is of sufficient size to allow apposition of the margins without excessive tension [2,4]. In the present case, however, the defect involving approximately 70% of the central tendon (4.5 × 5 cm) was too large for tension-free primary closure. The limited residual central tendon tissue resulted in significant tension on the sutures and incomplete defect closure. This finding is consistent with previous reports indicating that large central tendon defects pose a particular challenge for primary repair due to the relatively small contribution of the central tendon to the total diaphragmatic area [11,12].

Several techniques have been described for repairing large diaphragmatic defects when primary closure is not feasible. The ventral diaphragmatic advancement technique (VADT) involves incising the ventral attachments of the diaphragm and sliding it medially to achieve tension-free closure [11,12]. Hennet et al. described successful VADT after failure of a pericardial flap repair 7 months postoperatively, with an excellent outcome [11]. Perez-Bello similarly reported that VADT provided effective clinical resolution with minimal tension and a low complication rate [12]. Pericardial flap reconstruction has also been used but may carry a risk of late failure [10,11]. Synthetic mesh materials such as polypropylene have been employed but carry risks of infection, adhesion formation, and chronic inflammatory reactions [18,19].

In this case, cSIS was chosen primarily because SIS, as an acellular extracellular matrix, provides temporary mechanical support while promoting host cell migration, neovascularization, and site-specific tissue remodeling [13], and because SIS is gradually replaced by host tissue, potentially reducing the risk of long-term foreign body complications [13]. A further consideration—strictly at the theoretical level—was the allogeneic origin of cSIS. Porcine SIS contains the α-Gal epitope, which has been demonstrated throughout the full thickness of the scaffold and can bind naturally occurring anti-Gal antibodies [15]; because canines lack the α-Gal epitope, cSIS is theoretically free from this xenogeneic antigenic stimulus when implanted in canine patients. Yen et al. confirmed that removal of the α-Gal and Neu5Gc epitopes significantly reduces the inflammatory response to porcine SIS [16], and Lee et al. reported no signs of rejection or specific immune responses in two dogs treated with cSIS [14], as well as a more rapid CD8+ T cell decline in cSIS-implanted rats compared to polypropylene mesh [17]. It must be emphasized, however, that this immunological rationale remained entirely theoretical in the present case: no immunological, histological, or biomechanical evaluation of the repair site was performed. The clinical evidence base for cSIS in dogs remains limited to a single report of perineal herniorrhaphy in two dogs [14] and the present case. As limited context on alternative materials—without implying any direct comparability, given the differences in species, defect type, and clinical setting—pSIS has been used successfully to repair a congenital diaphragmatic hernia in a kitten [20] and has been evaluated for perineal herniorrhaphy in dogs [13,21]. No head-to-head comparative study between cSIS and these or other biomaterials has been performed, and no conclusion regarding the relative efficacy, mechanical strength, or degree of tissue integration of cSIS can be drawn from this single case.

The reherniation that occurred 42 days postoperatively in the present case warrants careful consideration. Importantly, the cSIS patch itself did not fail or break down; rather, the reherniation occurred at the marginal interface between the native diaphragm and the cSIS application site. It should be noted that the graft had been applied over the residual ventral defect that remained after incomplete primary closure—rather than over the entire original defect area including the dorsal primary suture line—and was anchored along the margin of this residual defect. Accordingly, the marginal interface at which dehiscence occurred corresponded to the junction between the graft and the native diaphragm along the edge of the residual defect, a location consistent with the marginal defect identified at the second surgery (Figure 6C). This pattern of marginal failure is consistent with the known mechanism of SIS-mediated tissue repair, in which the scaffold serves as a template for host cell infiltration and tissue deposition over a period of several weeks [13]. In the early postoperative period, the interface between the SIS patch and the host tissue may represent the weakest point of the repair, as complete integration and remodeling require time. Sandoval et al. demonstrated in a growing canine model that 4-ply SIS patches were associated with thinning, multiple defects, and visceral herniation in 3 of 5 surviving animals, whereas 8-ply patches were more durable with an 80% success rate [22]. Freitas and Hoddinott (2024) similarly emphasized that when SIS patches are used in juvenile animals, the thin semitransparent membrane observed at the graft site weeks to months postoperatively indicates that the remodeling process is still ongoing, and reassessment of the diaphragmatic repair should be considered during future abdominal surgeries [20]. These findings suggest that the thickness and layer composition of the SIS patch may influence repair strength and that marginal weakness at the patch–host tissue interface is a recognized potential complication.

Biologically, the graft–host junction is intrinsically vulnerable during the first weeks after implantation. SIS-mediated repair relies on gradual host cell infiltration, vascular ingrowth, and tissue deposition, and until this process matures, the interface between the scaffold and the surrounding tissue has not yet attained its eventual strength [13]; consistent with this, previous reports have noted that the thickness and layer composition of SIS patches influence repair strength and durability, and that remodeling is still ongoing weeks to months after implantation [20,23]. In the present case, this biological vulnerability was likely compounded by the configuration of the repair: the graft was joined edge-to-edge to the defect margin, without overlap onto the native diaphragm and without reinforcing buttress sutures, so that essentially the entire tensile load at the junction was borne by the primary suture line alone. An overlapping configuration, in contrast, would have distributed forces across a wider interface and provided a second mechanical barrier. Suture spacing, by contrast, is unlikely to have been the dominant factor, as the sutures were placed at a relatively close 2–3 mm interval in a simple interrupted pattern.

The environment in which this junction had to heal may also have contributed. Unlike relatively static fascial or perineal repair sites, the diaphragm undergoes continuous cyclical excursion with every breath, imposing repetitive tensile and shear loading on the patch–host junction many thousands of times per day; such cyclic stress could delay or oppose early graft integration and may precipitate marginal dehiscence even when the repair appears stable at rest. In addition, the dog’s activity was not restricted beyond routine postoperative care during the first weeks, so transient surges in intra-abdominal pressure—jumping, excitement, or vomiting—cannot be excluded as sources of acute loading across the immature interface, although the actual activity pattern was not documented. The graft bed itself may also have been less favorable than other anatomical sites: the central tendon is a relatively avascular, densely collagenous structure compared with the muscular portions of the diaphragm, and this limited local blood supply may have delayed host cell migration and vascular ingrowth, further prolonging the vulnerable early period of the marginal junction. Taken together, the observation that the cSIS patch remained intact and grossly incorporated at 42 days argues against primary scaffold failure and localizes the failure to the marginal interface—most likely the combined result of immature integration, the edge-to-edge fixation configuration, the cyclic mechanical environment of the diaphragm, and the modest vascular bed of the central tendon—although the relative contribution of each cannot be determined from a single case.

At the second surgery, tissue grossly consistent with newly formed host tissue (referred to here as “self-tissue”) was observed at the cSIS application site. Although this tissue was thin, it retained sufficient suture-holding integrity and was grossly continuous with the surrounding native diaphragm without a discernible boundary; on the basis of these intraoperative findings, the marginal defect could be closed by direct suturing without significant tension, effectively converting the challenging large defect repair into a manageable marginal defect closure. This gross observation is in line with the known biological behavior of SIS, which is gradually degraded and replaced by organized host connective tissue over a period of 4–8 weeks [22,24]. It should be noted, however, that active vascularization was not grossly identified on the surface of this tissue; while the absence of visible vessels does not exclude capillary-level vascularization, neovascularization at the repair site could not be assessed in this case and remains unconfirmed. Moreover, no biopsy was performed in this case; therefore, the precise cellular nature of the newly formed tissue, as well as host–cell remodeling and neovascularization at the repair site, could not be histopathologically verified, and statements regarding these processes in this report remain inferential. We acknowledge that histological sampling of this newly formed tissue at the second surgery would have substantially enhanced the mechanistic value of this report.

In retrospect, the 2-ply configuration of the cSIS patch may have contributed to the marginal failure. As described in the surgical methods, the 2-ply configuration was selected based on the surgeon’s intraoperative judgment that two layers would provide sufficient thickness and tension for a graft applied as reinforcement over a partially closed defect, rather than as a load-bearing bridge. This experience illustrates that the optimal number of graft layers for clinical PPDH repair remains undetermined for large defects, and that graft thickness should therefore be explicitly considered when planning cSIS reinforcement. It should also be noted that configurations of one or two layers have been used successfully in prior reports: double-layer SIS sheets were used to reconstruct a large chronic diaphragmatic defect in a kitten, with restoration of normal clinical function and no restriction of chest wall development [25]; a double-layer SIS patch closed full-thickness gastric defects in rats [26]; single-layer SIS supported regeneration of large (5 × 5 cm) fascial defects in dogs, with tissue resembling normal fascia at 12 weeks [27]; and SIS provides suture retention strength that has been judged adequate for the repair of soft tissue defects [28]. These observations, however, do not establish that two layers are optimal for clinical diaphragmatic repair, and no study has directly compared layer counts for this application. Notably, in the present case the graft was joined directly to the diaphragmatic defect edge in a simple interrupted pattern at 2–3 mm intervals without graft overlap beyond the margin and without reinforcing sutures; given that the reherniation occurred precisely at this patch–host interface, the use of prophylactic reinforcing (buttress) sutures at the marginal interface, or overlap of the graft beyond the defect margins, might have reduced the risk of marginal reherniation. It must be acknowledged that these observations and suggestions are retrospective reflections drawn from the present complication, not elements of a pre-designed surgical plan: at the time of the first operation, no established guideline or consensus existed regarding overlap or buttress reinforcement for SIS patches applied to diaphragmatic repair, and the surgeon judged that edge-to-edge fixation with closely spaced interrupted sutures would provide adequate strength for a graft serving as reinforcement. In this sense, the marginal failure itself is what generated these recommendations, and they are offered as lessons for future applications rather than as techniques that were knowingly omitted. These considerations, taken together, should inform future applications of cSIS for diaphragmatic repair; appropriately designed comparative studies of alternative biomaterials, layer configurations, and fixation techniques are warranted before specific recommendations can be made.

Limitations of this report include the retrospective nature of data collection, the lack of histopathological evaluation of the repair site, and the fact that in-hospital imaging follow-up was limited to 5 months after the second surgery; however, a telephone interview with the owner 3 years after the second surgery confirmed that the dog remained free of clinical signs, although imaging was not repeated at that time point. In addition, all ultrasonographic examinations were performed by a single examiner, and Doppler assessment was limited to qualitative color Doppler confirmation of blood flow; contrast-enhanced ultrasonography, and quantitative flow parameters (such as peak systolic velocity and resistive index) were not obtained or recorded, so inter-observer variability could not be assessed. CT was also not repeated prior to the second surgery, in order to avoid a separate anesthetic event for diagnostic imaging in a patient considered to be at relatively high anesthetic risk; consequently, the position and size of the recurrent hernia ring were not characterized preoperatively by cross-sectional imaging, and the anatomical evidence for the marginal mechanism of reherniation rests on the intraoperative findings obtained at the second surgery (Figure 6C), which did not allow objective preoperative comparison of the defect anatomy between the first and second surgeries. In addition, technical aspects of the graft fixation may have contributed to the marginal weakness that led to reherniation: although two layers were judged sufficient at the time of surgery, the graft was placed without overlap beyond the diaphragmatic margin and without reinforcing sutures, so the possibility that a thicker graft, graft overlap beyond the defect margin, or buttress sutures would have better withstood marginal dehiscence cannot be excluded; accordingly, the suggestions regarding graft overlap and buttress sutures raised in the Discussion represent retrospective reflections informed by this outcome, not elements of a pre-designed surgical protocol. Because no biopsy was obtained, descriptions of tissue remodeling and self-tissue formation in this report are based on gross intraoperative and ultrasonographic findings rather than histological confirmation; accordingly, the thin but sutureable quality of the presumed self-tissue, its gross continuity with the native diaphragm, and the absence of visible active vascularization—with the possibility of capillary-level vascularization remaining unevaluated—constitute gross assessments only. We acknowledge that histological sampling of this newly formed tissue at the second surgery would have substantially enhanced the mechanistic value of this report. Furthermore, this report provides no comparative data: the mechanical strength, suture-holding tension, and degree of patch integration of the repair were not measured, and no comparison with pSIS, canine fascia lata allograft, or synthetic mesh was performed. Accordingly, the immunological advantage of cSIS over pSIS discussed in this report should be regarded as an untested theoretical hypothesis rather than a finding of this study, and statements about the relative performance of cSIS versus other biomaterials await appropriately designed comparative studies.

## 4. Conclusions

In this case, cSIS patch reinforcement appeared to serve as a feasible adjunct to primary repair for a large PPDH defect that could not be completely closed with sutures alone. At the second surgery, gross intraoperative findings suggested new tissue formation at the cSIS application site, which enabled successful secondary repair of a marginal reherniation; however, because no biopsy was obtained, the cellular nature of this tissue and the underlying host–cell remodeling and neovascularization could not be histopathologically confirmed, and such descriptions in this report remain based on gross and imaging findings. Moreover, as these observations are drawn from a single case without comparative or biomechanical data, they should be regarded as preliminary, the theoretical immunological advantage of cSIS over alternative biomaterials remains untested in this setting, and no conclusions can be drawn regarding the relative performance of cSIS compared with other biomaterials. The marginal interface between the cSIS patch and the native diaphragm may represent the weakest point of the repair in the early postoperative period, and close postoperative monitoring, including serial thoracic radiography and ultrasonography, is recommended to detect marginal reherniation early. The optimal number of SIS graft layers for clinical diaphragmatic repair remains to be determined, and marginal overlap techniques may improve outcomes in future applications. Further studies with larger case numbers, histopathological and biomechanical evaluation, and longer follow-up periods are needed to establish the safety, efficacy, and optimal application technique of cSIS for diaphragmatic hernia repair in dogs.

## Figures and Tables

**Figure 1:**
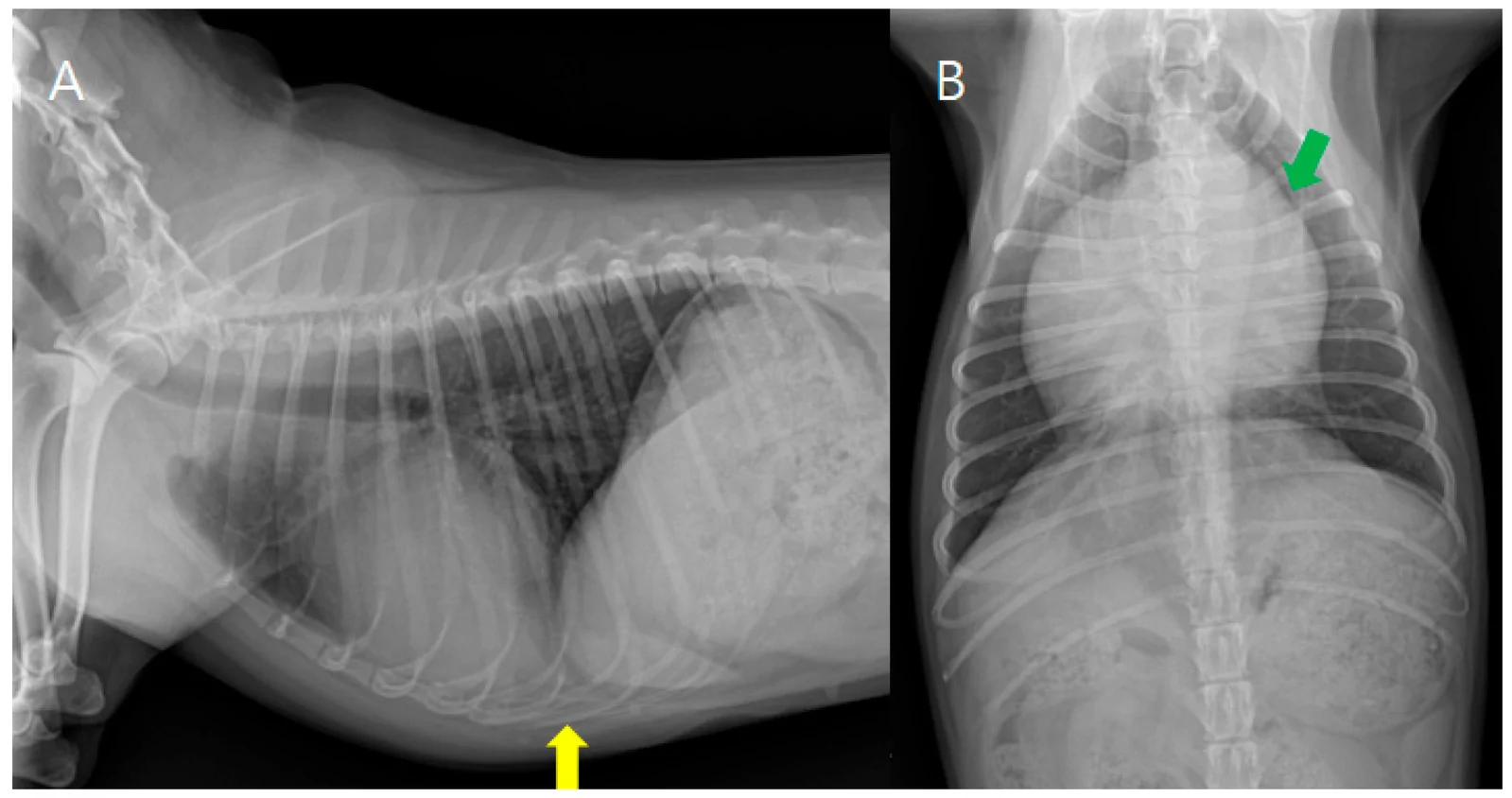
Preoperative thoracic radiographs. (**A**) Right lateral view showing a large, globoid cardiac silhouette with a poorly defined caudal cardiac border and diaphragmatic outline (yellow arrow), and a soft-tissue opacity in the caudoventral region of the cardiopericardial silhouette, consistent with the presence of abdominal viscera within the pericardial sac. (**B**) Ventrodorsal view demonstrating leftward displacement (green arrow) of the cardiac apex. These findings are consistent with peritoneopericardial diaphragmatic hernia.

**Figure 2:**
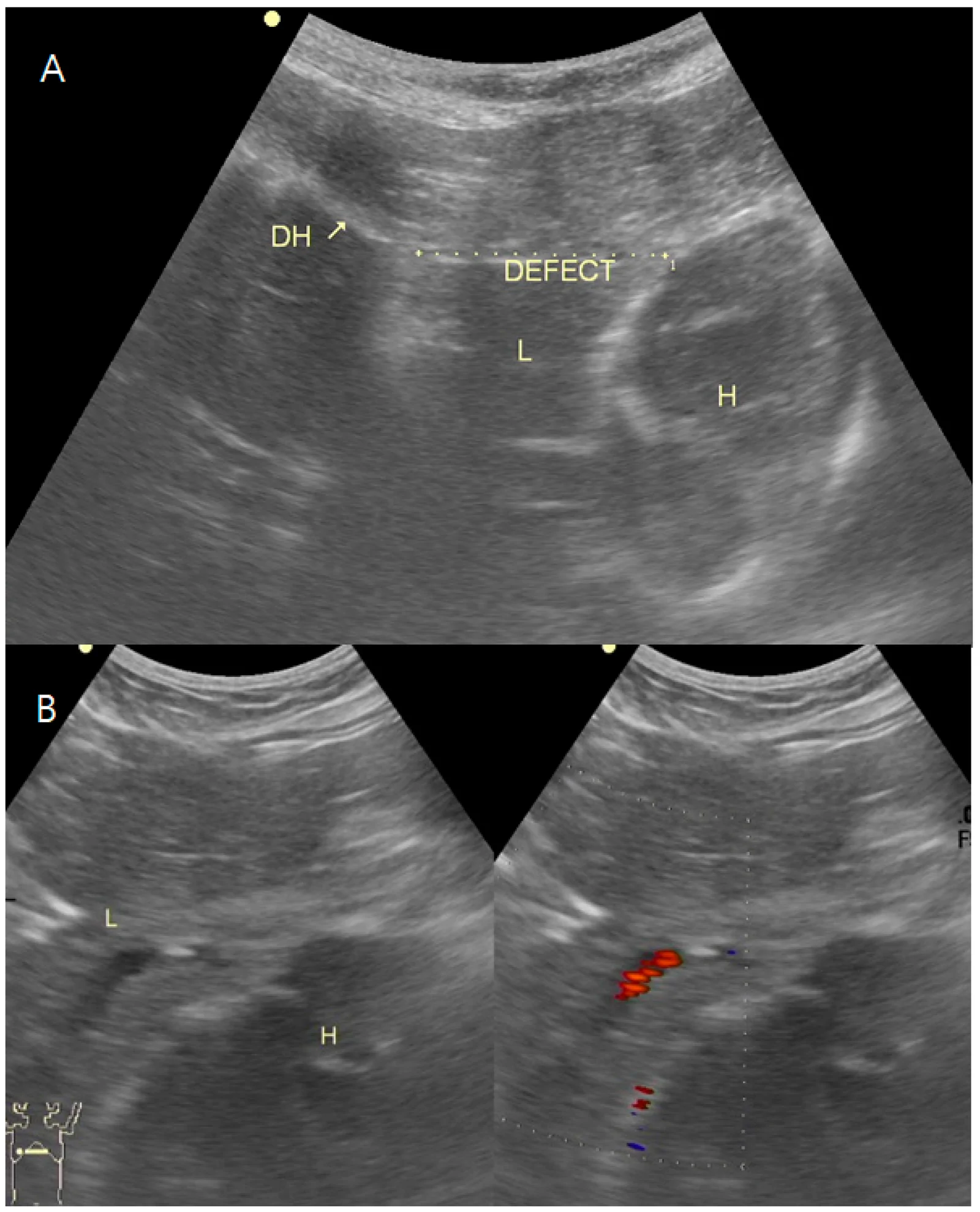
Preoperative abdominal ultrasonography images. (**A**) B-mode ultrasonography showing a liver lobe herniated through the diaphragmatic defect into the pericardial sac. (**B**) Color Doppler ultrasonography confirming blood flow within the herniated liver lobe. DH = diaphragm; L = liver; H = heart.

**Figure 3:**
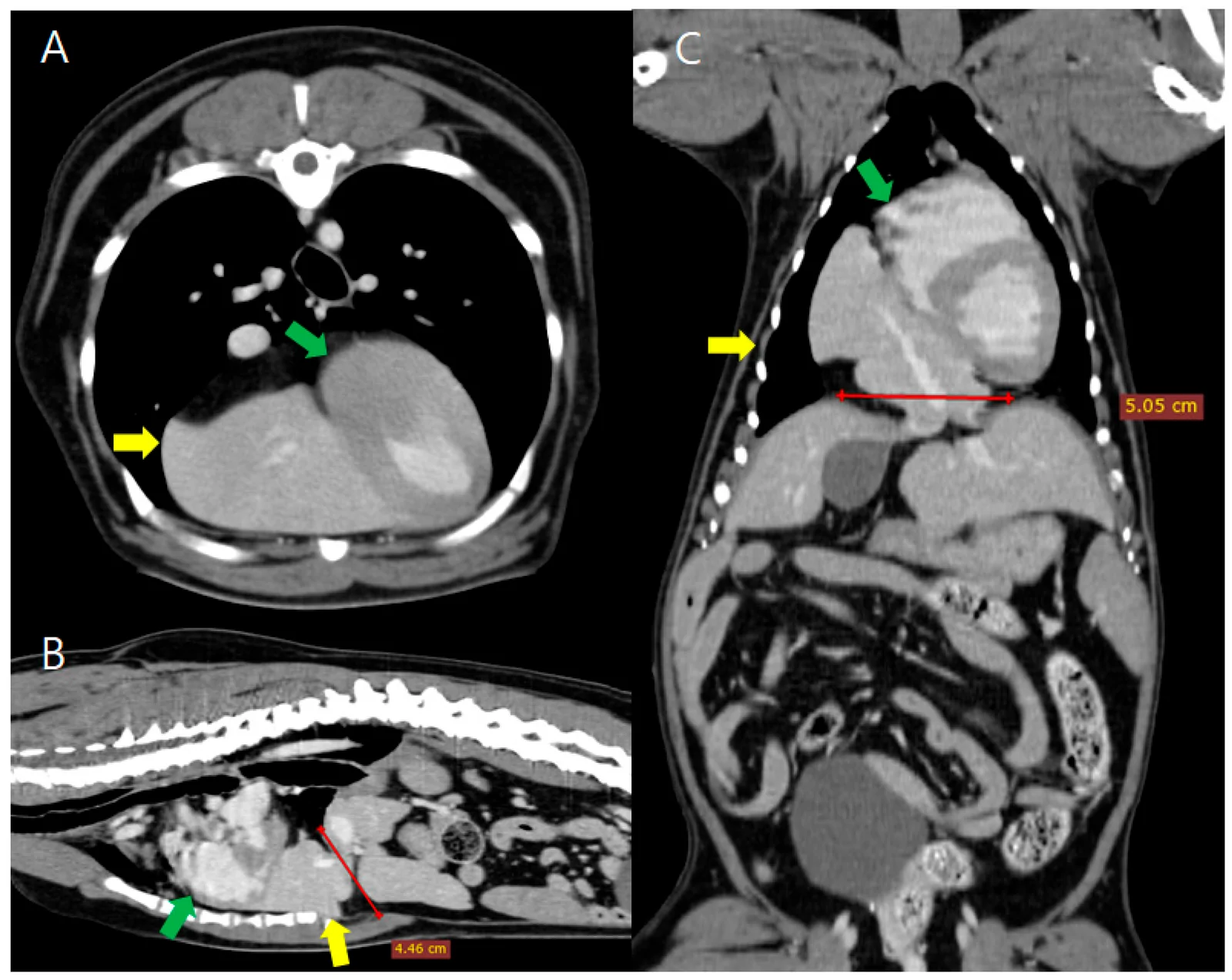
Preoperative contrast-enhanced thoracic and abdominal CT images with multiplanar reconstruction. (**A**) Transverse view showing the herniated liver lobe within the pericardial sac and leftward displacement of the heart. (**B**) Sagittal view demonstrating the craniocaudal extent of the herniation. (**C**) Dorsal view illustrating the overall morphology and lateral extent of the herniated organs within the pericardial sac. Red line: location and length of the hernia; yellow arrow: liver lobe herniated into the pericardial sac; green arrow: heart displaced by the mass effect of the herniated organs.

**Figure 4:**
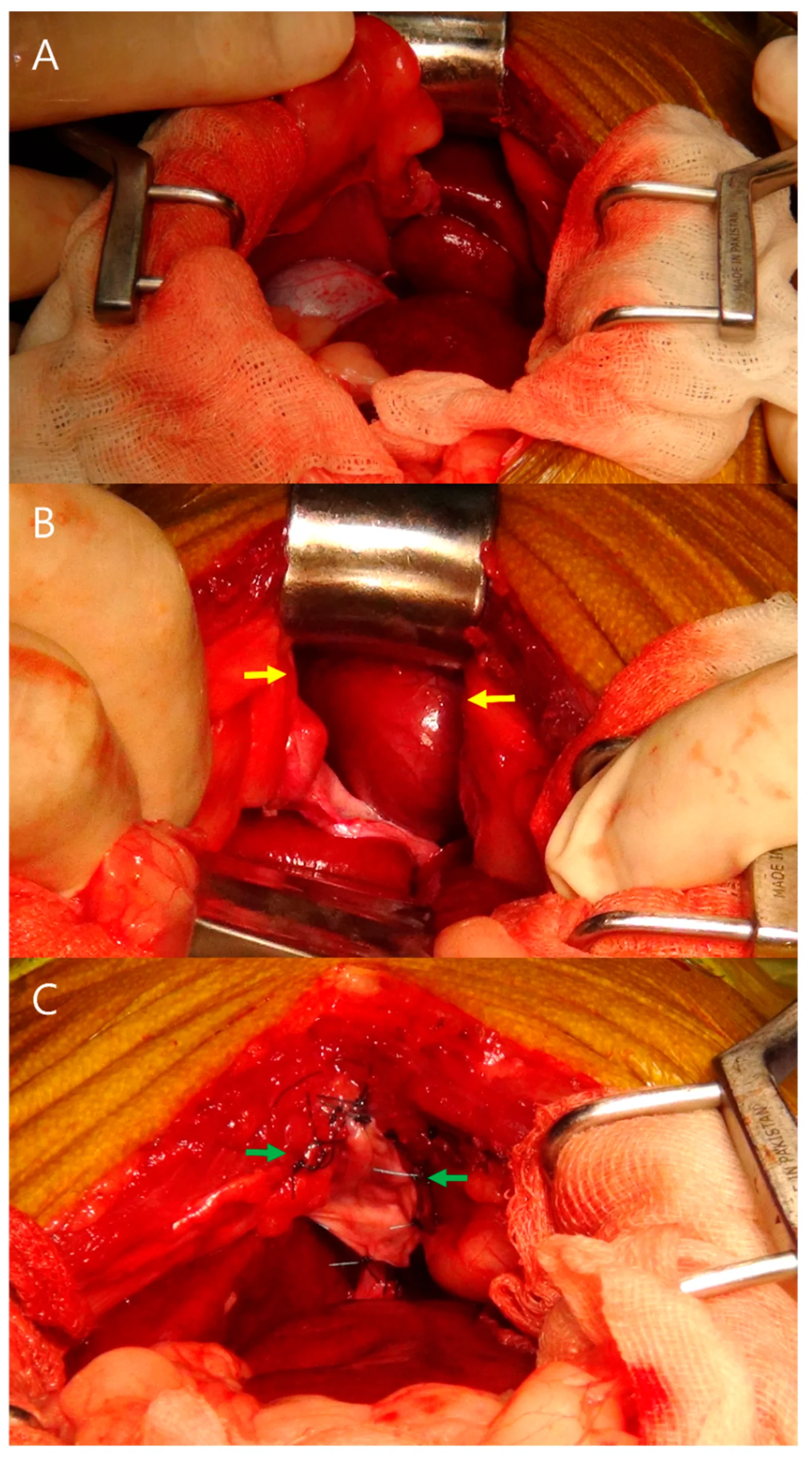
Intraoperative photographs of the surgical repair. (**A**) Herniated left medial and quadrate liver lobes within the pericardial sac. (**B**) The diaphragmatic defect at the central tendon after reduction of the herniated organs, measuring approximately 4.5 × 5 cm; primary closure was attempted but could not be completely achieved due to excessive tension. Yellow arrow: diaphragmatic defect. (**C**) The 2-ply canine small intestinal submucosa (cSIS) patch sutured over the residual defect. Green arrow: 2-ply cSIS graft.

**Figure 5:**
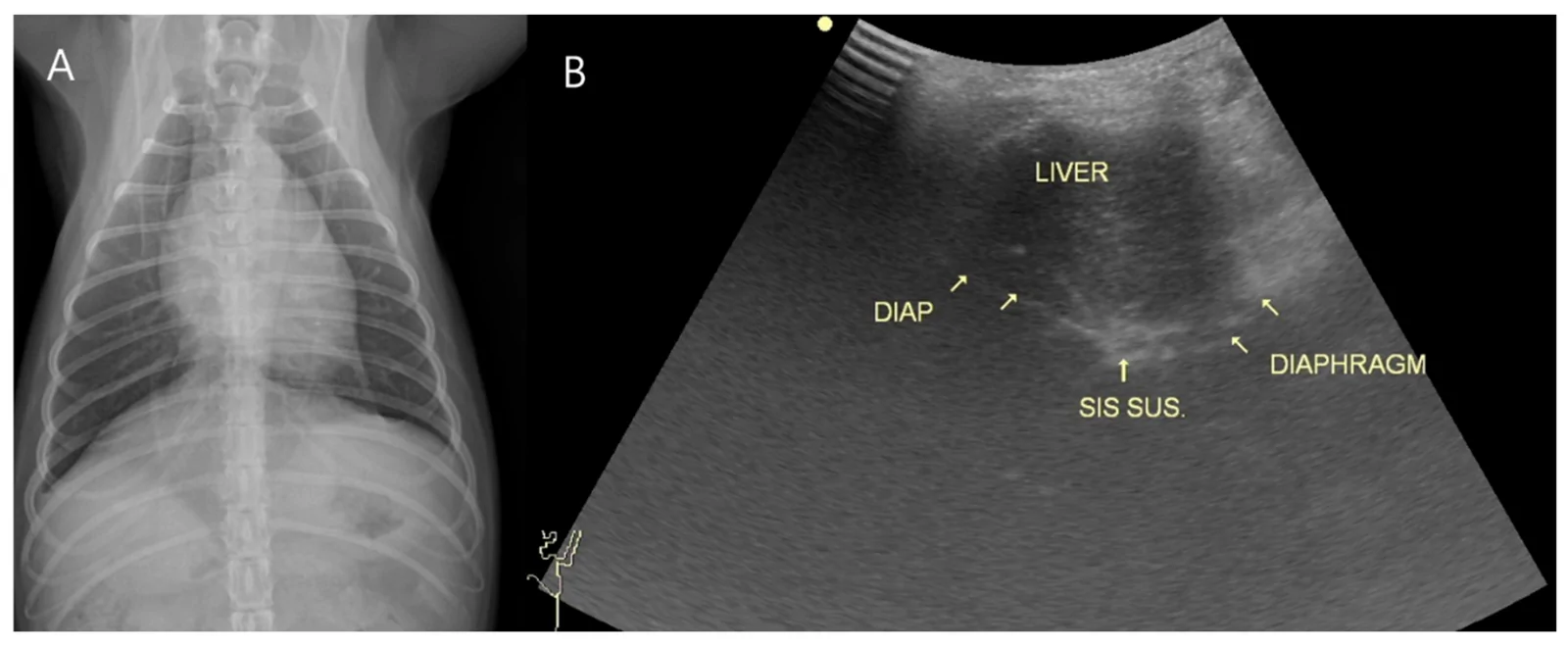
Postoperative follow-up imaging at the time of suture removal (11 days postoperatively). (**A**) Postoperative VD thoracic radiograph demonstrating resolution of the globoid cardiomegaly and a normal cardiac silhouette. (**B**) Abdominal ultrasonography confirming the cSIS patch is well maintained at the defect site with no evidence of reherniation. DIAP = diaphragm; SIS SUS = cSIS (suspected application site).

**Figure 6:**
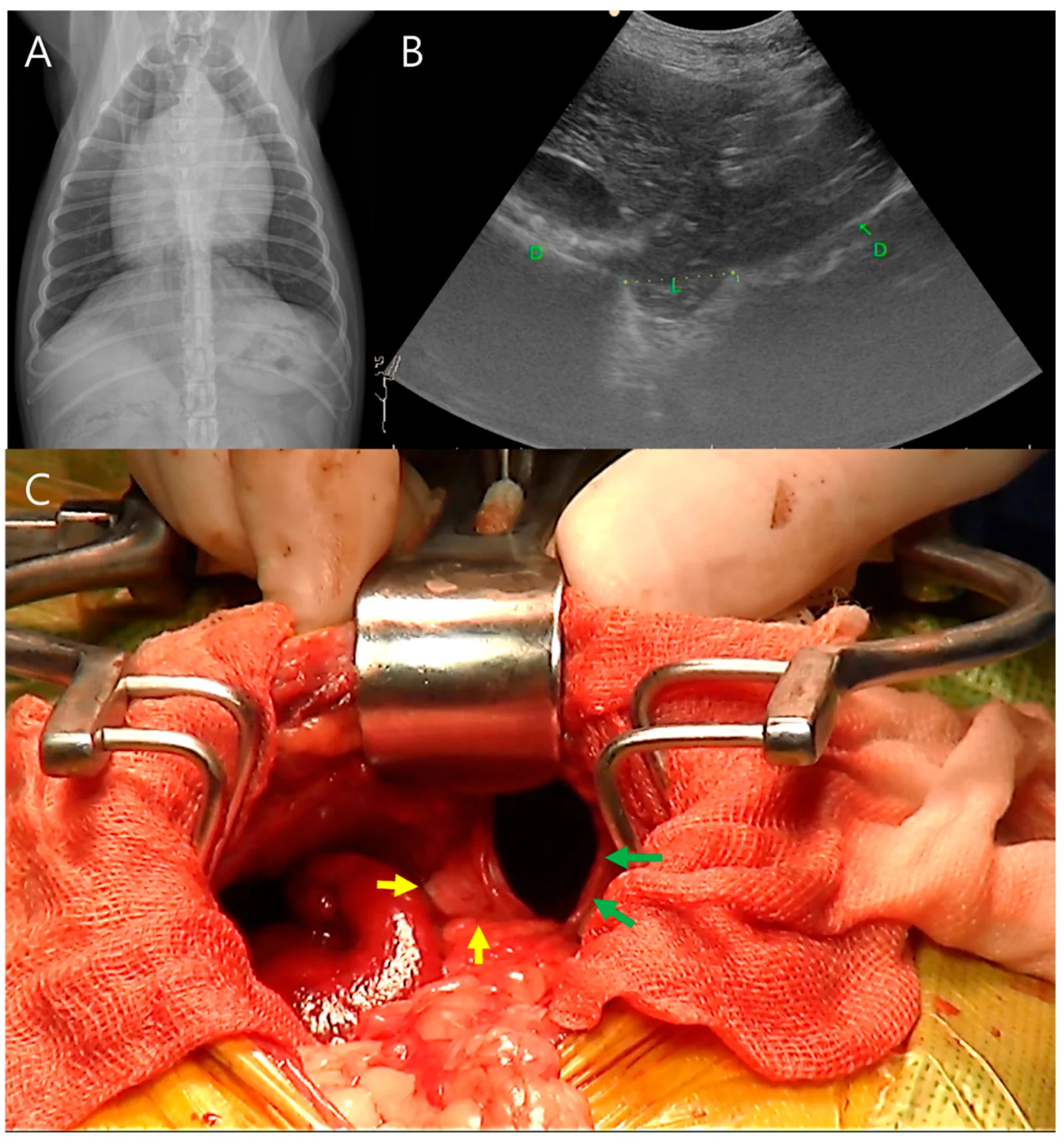
Imaging and intraoperative findings at the time of reherniation (42 days postoperatively). (**A**) Ventrodorsal thoracic radiograph showing recurrence of cardiomegaly with an increased cardiac silhouette long axis measurement (90.8 mm), consistent with reherniation. (**B**) Abdominal ultrasonography demonstrating herniation of a liver lobe into the pericardial sac. D = diaphragm; L = liver lobe herniated into the pericardial sac. (**C**) Intraoperative view during the second surgery. Yellow arrow: presumed self-tissue formed at the site of previous cSIS application. Green arrow: marginal defect (dehiscence) at the interface between the cSIS patch and the host diaphragmatic tissue, through which reherniation occurred.

**Figure 7:**
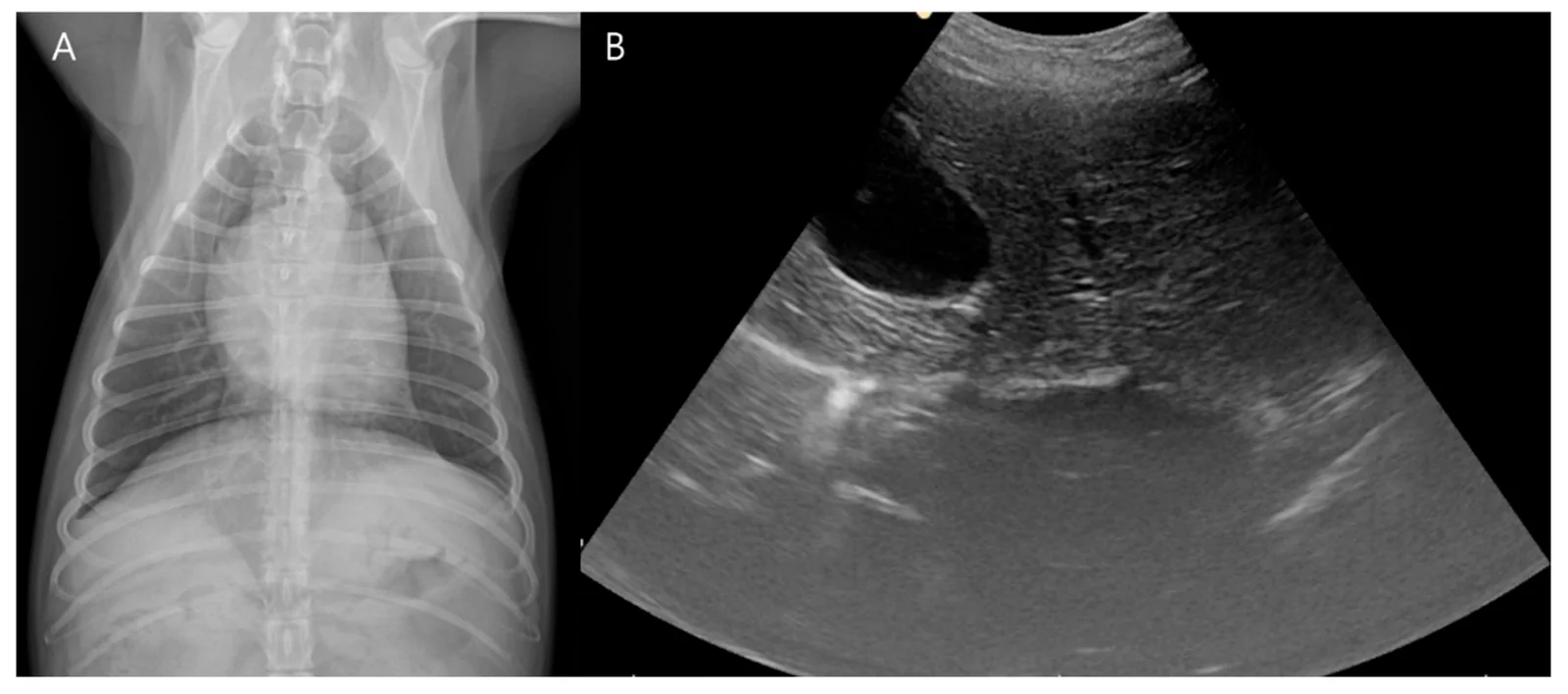
Long-term follow-up imaging 5 months after the second surgery. (**A**) Ventrodorsal thoracic radiograph showing a normal cardiac silhouette with no evidence of cardiomegaly or reherniation. (**B**) Abdominal ultrasonography confirming an intact diaphragm with no evidence of reherniation.

**Table 1:** Hematologic and serum biochemical findings at initial presentation.

Parameter	Measured Value	Reference Interval
WBC	9.6 × 10^3^/μL	6.0–17.0 × 10^3^/μL
RBC	7.32 × 10^6^/μL	5.5–8.5 × 10^6^/μL
Hemoglobin	17.1 g/dL	12.0–18.0 g/dL
PCV	48.8%	37–55%
MCV	66.7 fL	60–77 fL
MCH	23.4 pg	19.5–25.5 pg
MCHC	35%	32–37%
Platelets	557 × 10^9^/L	150–500 × 10^9^/L
BUN	16 mg/dL	7–27 mg/dL
Creatinine	0.8 mg/dL	0.5–1.8 mg/dL
ALT	59 U/L	10–125 U/L
AST	26 U/L	15–66 U/L
ALP	52 U/L	23–212 U/L
Glucose	98 mg/dL	74–143 mg/dL
Total protein	6.4 g/dL	5.2–8.2 g/dL
Albumin	3.8 g/dL	2.3–4.3 g/dL

## Data Availability

The original contributions presented in this study are included in the article. Further inquiries can be directed to the corresponding author.
